# Cockayne syndrome

**DOI:** 10.4103/0972-2327.41884

**Published:** 2008

**Authors:** Firosh Khan, Thomas Chemmanam, P. S. Mathuranath

**Affiliations:** Department of Neurology, Sree Chitra Tirunal Institute for Medical Sciences and Technology, Trivandrum, India

## Case Report

**Figure 1:**
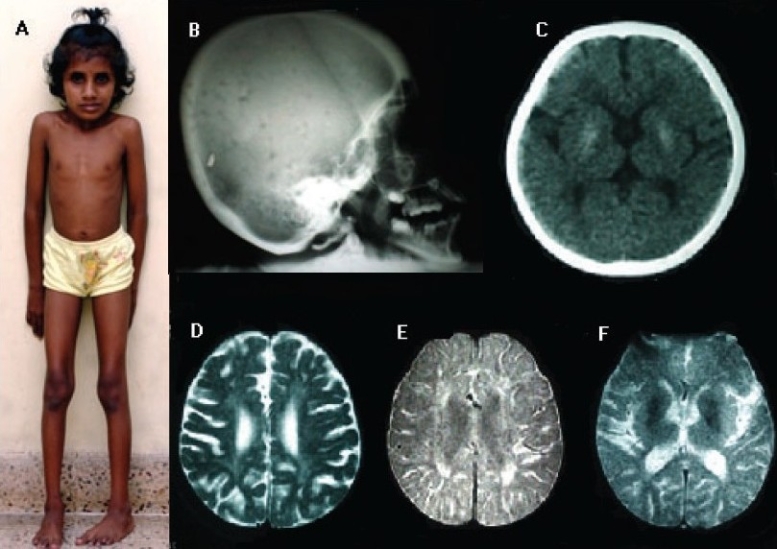
**A:** Emaciated, dwarf child with progeric face (‘cachectic dwarfism’). Also note the foot dystonia; **B:** Lateral skull x-ray showing calvarial thickening; **C:** Axial CT scan (at 5 years of age) showing bilateral basal ganglia calcifications; **D, E,** and **F:** Axial MRI scans of the brain (T2WI, proton density image, and gradient spin-echo image, respectively) showing generalized atrophy, symmetrical white matter hyperintensities, and basal ganglia calcification

A 9-year-old girl, born to nonconsanguineous parents, with normal antenatal and neonatal periods, was hospitalized for the assessment of a global developmental delay. She had had a tonic–clonic seizure at 2 years of age and subsequently had poor feeding and progressive emaciation. By the age of five, she had developed decline in speech, unsteadiness of upper limbs, worsening of gait with posturing of the feet, and toe walking. Examination showed a short-statured, emaciated girl with a progeric face and microcephaly [Figure 1A]. She had moderate mental retardation, relatively brisk deep tendon reflexes, upper limb incoordination, and foot dystonia. Fundoscopy showed a salt-and-pepper appearance, with macular thickening and pigment mottling. Routine blood tests were normal. Review of the brain CT scan done at 5 years of age showed bilateral basal ganglia calcifications [Figure 1C]. A detailed skeletal survey showed a thickened calvarium [Figure 1B] and bilateral short fourth metatarsals. An MRI scan of the brain revealed generalized cerebral and cerebellar atrophy, with diffuse white matter hyperintensities [Figures 1D and 1E], and hypointensities of the lentiform nuclei in T2-weighted images, which were consistent with calcifications [Figure 1F]. Electroencephalogram and nerve conduction studies were normal.

## Discussion

The salient observations in our patient were developmental delay/regression, poor food intake, growth failure, emaciation, microcephaly, progeria, dystonia, ataxia, retinal pigmentary changes, basal ganglia calcification, and brain parenchymal atrophy with white matter signal changes, and calvarial thickening. All these features are characteristic of the Cockayne syndrome (CS) and the patient satisfied the clinical criteria for classical Cockayne syndrome.[1] The Cockayne syndrome is an autosomal recessive, DNA repair-deficient disorder, presenting with a variety of somatic and neurological manifestations.[2,3] The syndrome spans a spectrum but can be reasonably classified into four distinct types.[1] There are clinical diagnostic criteria described for each type[1]; the four types are as follows:

1. CS type I: The ‘classic’ form with growth and developmental abnormalities beginning in the initial two years of life. There is progressive impairment of vision, hearing, and central and peripheral nervous system function, leading to severe disability. Death typically occurs in the first or second decade.
2. CS type II: Also known as the cerebro-oculo-facial skeletal syndrome (COFS) or ‘connatal’ Cockayne syndrome. This is a severe form, with growth failure present at birth and little or no postnatal neurological development. Ocular and spinal anomalies may be present, and affected children typically die by around seven years of age.
3. CS type III: A rare, comparatively mild form characterized by a relatively later onset; there may be essentially normal growth and cognitive development.
4. Xeroderma pigmentosum-Cockayne syndrome (XP-CS): An overlap syndrome having features of both.[4]

The diagnosis is supported by molecular genetic tests of *ERCC6* and *CKN1* genes linked to the syndrome as well as by DNA repair assays.[5] However, these tests are still not widely available[5] and so the prompt identification of the clinical syndrome[1] is crucial for a proper diagnosis. The typical neuroimaging features, as in our case, can clinch the diagnosis.
